# A Case of OHVIRA (Obstructed Hemivagina and Ipsilateral Renal Anomaly) Syndrome Diagnosed After Signs of Infection During Pregnancy

**DOI:** 10.7759/cureus.69823

**Published:** 2024-09-20

**Authors:** Toyofumi Hirakawa, Daichi Urushiyama, Masamitsu Kurakazu, Fusanori Yotsumoto

**Affiliations:** 1 Department of Obstetrics and Gynecology, Faculty of Medicine, Fukuoka University, Fukuoka, JPN

**Keywords:** herlyn-werner-wunderlich syndrome, intrauterine infection, ohvira syndrome, unilateral renal agenesis, uterine abnormality

## Abstract

Obstructed hemivagina and ipsilateral renal anomaly (OHVIRA) syndrome is a rare group of disorders that affects women at all stages of life. These disorders can elicit symptoms such as menstrual molimina and dysmenorrhea during puberty; miscarriage, premature birth, and infertility during childbearing age; and purulent discharge during menopause and old age. In this study, we report our experience with OHVIRA syndrome, which was diagnosed during childbearing age when the patient showed signs of infection during pregnancy. The patient was a 28-year-old female diagnosed with OHVIRA syndrome during pregnancy who had previously undergone a cesarean section. Despite having a normal prenatal period, the patient experienced lower abdominal pain at 27 weeks gestation, prompting urgent hospitalization. Clinical signs suggested chorioamnionitis; however, the amniocentesis results were negative. Computed tomography and magnetic resonance imaging demonstrated unilateral renal agenesis and a duplicated uterus, characteristics of OHVIRA syndrome, in addition to a uterine infection on the non-pregnant side. Antibiotic treatment enabled the pregnancy to continue until an emergency cesarean section was performed at 31 weeks. Surgery confirmed OHVIRA syndrome with incomplete obstruction. This case highlights the challenges that can arise during the perinatal period due to a partially obstructed duplicated uterus. It emphasizes the importance of performing ultrasonographic renal evaluations when assessing uterine malformations, as this can aid in early detection and effective management of women's health, especially during the perinatal period. Despite its rarity, it is crucial for healthcare providers to be aware of OHVIRA syndrome during clinical interventions.

## Introduction

The spectrum of disorders involving uterine duplication (manifesting as a duplicated or bicornuate uterus), paracervical cysts, and pathological lateral renal aplasia includes Wunderlich syndrome, Herlyn-Werner syndrome, and obstructed hemivagina and ipsilateral renal anomaly (OHVIRA) syndrome. These syndromes are caused by defective fusion of the left and right Müllerian ducts, anomalies of the Wolffian ducts, and lack of development in the unilateral Wolffian duct. These disorders are rare and can affect a woman's quality of life at all stages: menstrual molimina and dysmenorrhea during puberty; miscarriage, premature birth, and infertility during sexual maturity; and purulent discharge during menopause and old age.

Wunderlich and OHVIRA syndromes can often be identified in early puberty due to the accumulation of menstrual blood in the paracervical cyst on the affected side, eliciting symptoms similar to menstrual pain. However, the clinical manifestations of uterine duplication often remain unrecognized until adulthood, potentially resulting in missed diagnoses. Pregnancy may occur without knowledge of the underlying syndromes; however, it is considered a high-risk condition and can lead to perinatal complications. Although sporadic reports on these syndromes have emerged due to the use of imaging techniques such as ultrasonography and magnetic resonance imaging (MRI), studies on pregnancy and perinatal outcomes in women with OHVIRA syndrome are scarce. In this paper, we will elaborate on our experience with OHVIRA syndrome, which was diagnosed during pregnancy due to signs of infection.

## Case presentation

The patient was a 28-year-old woman. At 27 weeks of gestation, she sought medical care due to sudden lower abdominal pain and genital bleeding. She was transferred to our medical facility. The patient experienced menarche at the age of 14, with a regular 28-day cycle lasting 7 days. She had intermittent malodorous discharge, which was closely monitored. Ten years ago, she underwent surgery to remove a cyst from the right vaginal wall, which revealed an abnormal uterine shape. She had two pregnancies and one delivery. Three years ago, the patient underwent an elective cesarean section due to breech presentation, with no complications during the pregnancy. During the first pregnancy, her previous physician carefully reviewed her medical history and did not detect a partially obstructed bicervical bicornuate uterus during an internal examination. Upon admission, the patient had a temperature of 37.4°C and a pulse rate of 88 beats per minute. A colposcopic examination revealed a brownish discharge with an odor. Only one cervix was visible, but a small fistula was found at the 10 o'clock position on the vaginal wall. There was no cyst on the right vaginal wall. Hematological tests showed an elevated inflammatory response, with a white blood cell count of 16,000/µL and a C-reactive protein level of 2.07 mg/dL. The results of blood tests performed at admission are presented in Table 1.

**Table 1:** Hematological tests performed at admission

Investigation	Result (normal range)
Hemoglobin	8.5 (11.6–14.8 g/dL)
White blood cell count	16.0 (3.3–8.6 × 10^3^/μL)
Platelet count	416 (158–348 × 10^3^/μL)
Hematocrit level	28.1 (35.1–44.4%)
Mean cell volume	81.2 (83.6–98.2 fL)
Mean cell hemoglobin level	24.6 (27.5–33.2 pg)
Mean cell hemoglobin concentration	30.2 (31.7–35.3 g/dL)
Neutrophil ratio	87.7 (50–70%)
Lymphocyte ratio	8.7 (20–40%)
Monocyte ratio	2.9 (1–6%)
Eosinophil ratio	0.4 (1–5%)
Basophil ratio	0.3 (0–1%)
Prothrombin time	11.9 (9.8–12.1 s)
International normalized ratio	1.02 (0.8–1.1)
Activated partial thromboplastin time	24.6 (24.0–34.0)
Sodium level	136 (138–145 mmol/L)
Potassium level	3.6 (3.6–4.8 mmol/L)
Urea nitrogen	7 (8–20 mg/dL)
Enzymatic creatinine	0.54 (0.46–0.79 mg/dL)
Calcium level	8.5 (8.8–10.1 mg/dL)
Albumin level	2.8 (4.1–5.1 g/dL)

**Figure 1:**
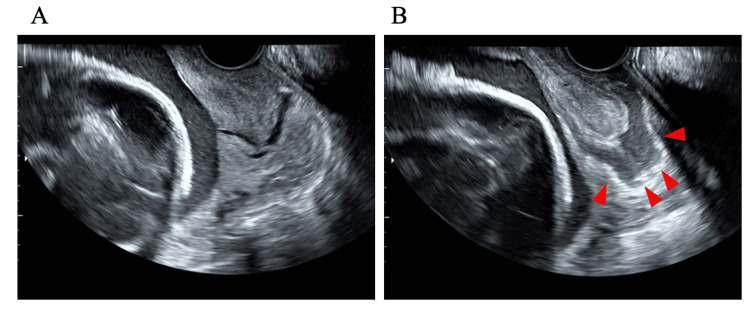
Transvaginal ultrasonography. (A) Normal uterine cervical line. (B) Hypoechoic area on the right lateral side of the cervical line.

Transvaginal sonography revealed a cervical length of 44 mm and a hypoechoic region measuring 30 x 17 mm near the chorion on the right side of the cervical line (Figure 1). The fetal heart rate contraction diagram showed uterine contractions occurring every five to six minutes. 

In response to signs of infection, antimicrobial therapy with tazobactam/piperacillin (TAZ/PIPC) and azithromycin (AZM) was initiated, along with magnesium sulfate to reduce uterine contractions. Hematological analyses conducted within six hours of admission revealed worsening of the inflammatory response. The patient's symptoms aligned with the diagnostic criteria for clinical chorioamnionitis, as outlined by Lencki et al. [1]. To confirm the diagnosis, amniocentesis was performed. However, the cell count in the amniotic fluid was 1/µL, the lactate dehydrogenase level was 209 U/L, and the glucose concentration was 42 mg/dL, all of which indicated a negative result for chorioamnionitis.

**Figure 2:**
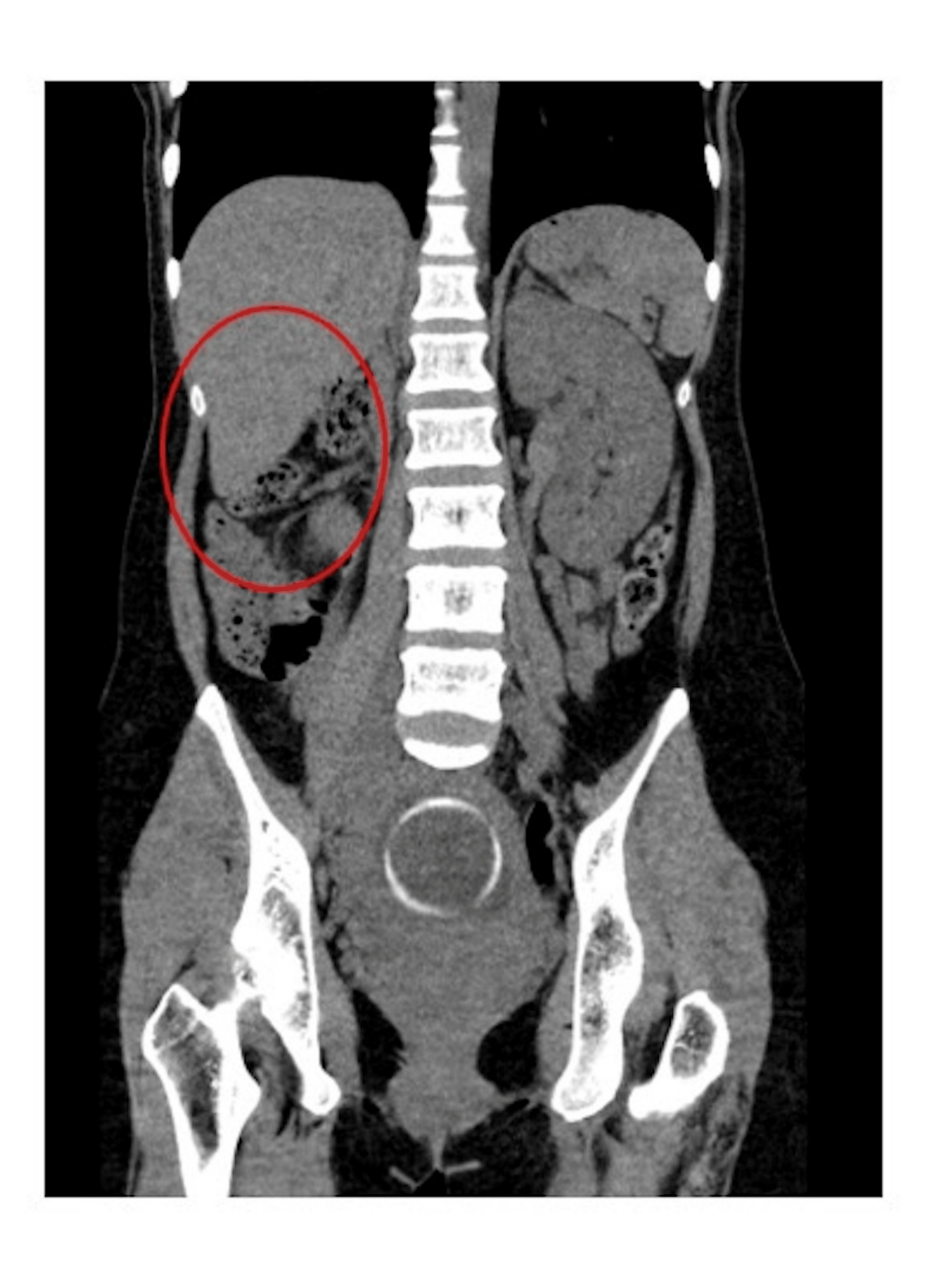
Computed tomography scan (coronal section) showing an absent right kidney (red circle).

Due to the proximity of a hypoechoic region near the previous cesarean section scar, there were concerns about a potential uterine rupture. Therefore, computed tomography (CT) of the abdomen was conducted, which ruled out uterine rupture but revealed a deficiency in the right kidney (Figure 2).

**Figure 3:**
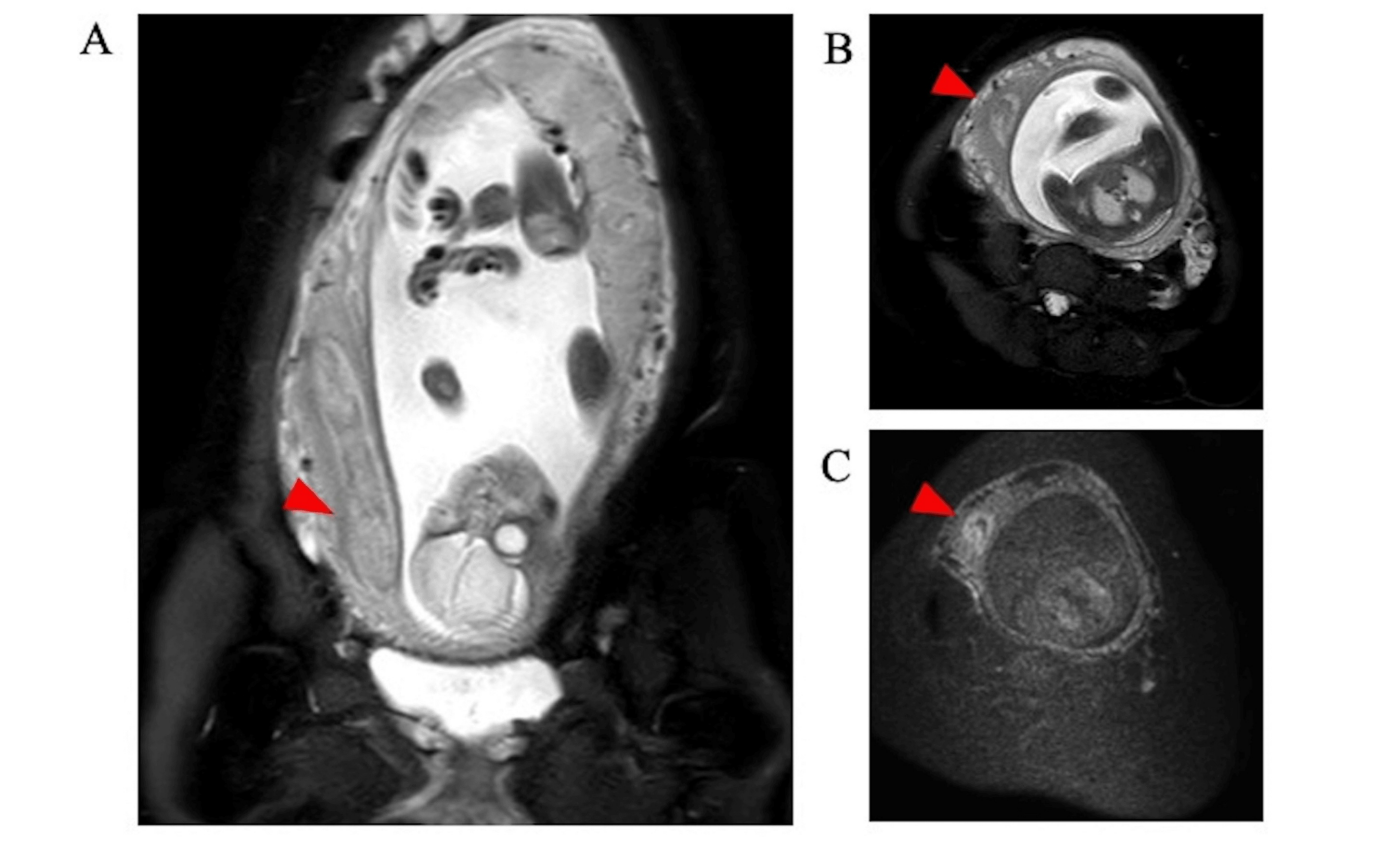
Magnetic resonance imaging of the abdomen. (A) T2-weighted image (coronal section). (B) T2-weighted image (horizonal section). (C) Diffusion-weighted image (horizonal section. The red arrow suggests a space-occupying lesion in the right uterus.

Subsequently, MRI of the pelvic region revealed an anatomical anomaly: a duplicated uterus with an amniotic cavity within the left uterus. The right uterus was stretched, and a space-occupying lesion was found. The lesion presented with a high signal intensity on T2-weighted images (T2WI) and, to some extent, an elevated signal intensity on diffusion-weighted imaging (DWI), indicating an infected endometrium (Figure 3). In the cervical region, a normal cervical canal connected to the amniotic cavity was noted. Additionally, a cervical canal-like structure was present on the right side of the typical cervical canal. This structure had slight thickening compared to the standard cervix and presented with a high signal intensity on T2WI, indicating infection. This area also showed a parallel high signal on DWI, further signifying infection (Figure 3).

Based on these findings, the clinically suspected diagnosis was OHVIRA syndrome, characterized by a duplicated uterus with a blind-angle uterus on one side and an ipsilateral kidney defect, along with infection in the non-gravid right uterus.

**Figure 4:**
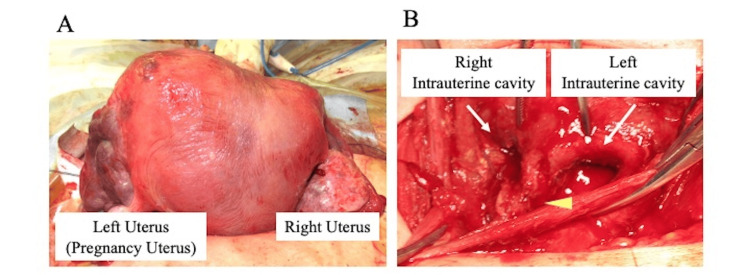
Intraoperative images (cesarian section) (A) Intestinal surface of the uterus. (B) Intrauterine cavity. The yellow arrowhead indicates the septal defect of the uterus.

Continuous antimicrobial therapy was initiated, which gradually reduced the inflammation, and uterine contractions were effectively managed with uterine contraction suppressants. However, during the 31st week of gestation, there was a resurgence of the inflammatory response along with an increase in uterine contractions. A tender cyst on the right vaginal wall was discovered during a vaginal examination. Attempts to drain the cyst by aspirating its contents were unsuccessful. As labor progressed, the cystic contents were drained through a fistula in the right vaginal wall. An emergency cesarean section was performed, resulting in the delivery of a female neonate weighing 1,692 g. During surgery, the uterus was found to be divided by a septum, with a connection between the compartments at the lower aspect (Figure 4).

A Neraton catheter was inserted through the fistula in the right vaginal wall, revealing contact with the right cervix. No abnormalities were found in the bilateral adnexa. Pathological analysis of the cyst's contents showed necrotic decidual membrane tissue. Following the surgery, the inflammation subsided rapidly, and the patient was discharged on the fifth day post-procedure.

## Discussion

Wunderlich syndrome, Herlyn-Werner syndrome, and OHVIRA syndrome are a group of disorders characterized by uterine duplication (resulting in a duplicated or bicornuate uterus), paracervical cysts, and pathological lateral renal aplasia. These disorders have a significant impact on women's healthcare from puberty to adulthood, including pregnancy and childbirth. Herlyn-Werner syndrome was first described by Herlyn and Werner in 1971 and is characterized by a duplicated uterus, Gartner's duct cyst, and aplasia of the affected kidney [2]. Wunderlich syndrome, reported in 1976, is characterized by a duplicated uterus, a unilateral blind-angle uterus, and aplasia of the affected kidney [3]. Early diagnosis of these syndromes is possible shortly after menarche, as menstrual blood accumulates in the paracervical cyst on the affected side, eliciting symptoms similar to menstrual pain. Nevertheless, some cases have been reported without explicitly categorizing them as Herlyn-Werner-Wunderlich syndrome [4].

In 2007, OHVIRA syndrome was documented as a spectrum of disorders featuring a duplicated uterus and vagina, unilateral vaginal closure, and aplasia of the affected kidney [5]. Since then, the classification of OHVIRA syndrome follows the taxonomy proposed by Rock and Jones [6]. The complete obstructive type is the most common, accounting for over half of the cases, and presents with progressive dysmenorrhea due to uterine and vaginal retention on the occluded vaginal side. Symptoms usually appear after menarche, and surgical intervention is necessary. The incomplete obstructive type involves communication between the right and left vagina and uterus, allowing menstrual blood to flow through this communication. Consequently, dysmenorrheic symptoms are mild and are often diagnosed in adulthood.

Distinguishing among these syndromes solely through imaging studies, such as ultrasonography and MRI, is challenging. Thus, histopathological differentiation is crucial for a definitive diagnosis due to the similarity in clinical symptoms. Herlyn-Werner syndrome is characterized by cuboidal, low columnar, or distinct ciliated epithelium derived from Gartner's duct cyst on the hematoma side. In contrast, Wunderlich syndrome, characterized by a unilateral blind-angle uterus, exhibits high columnar epithelium derived from cervical glands on the hematoma side. OHVIRA syndrome, characterized by duplicated vaginal closure, features multilayered squamous epithelium on the septum on both the hematoma side and the contralateral side. However, there are instances where histopathological confirmation is elusive, and cases have been reported where the nomenclature does not align with the histopathological features, suggesting potential overlap between these syndromes. Nevertheless, from an embryological perspective, these syndromes are considered nearly identical, limiting the clinical significance of strict distinctions. Consequently, recent trends lean toward collectively referring to these disorders as OHVIRA syndrome in a broader sense, without categorical differentiation [7].

**Figure 5:**
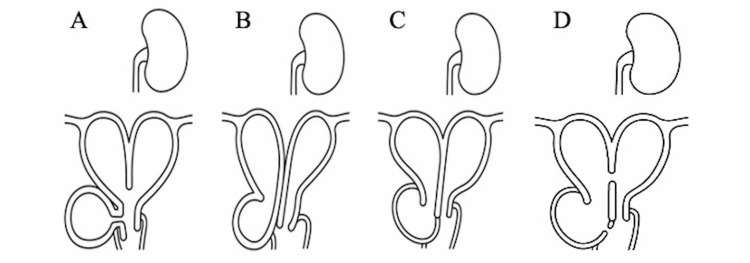
Schema of (A) Wunderlich syndrome, (B) Herlyn–Werner syndrome, (C) OHVIRA syndrome, and (D) the situation in the present case.

The identification or exclusion of a traffic-duplicated uterus is of significant clinical importance due to its impact on the patient's presentation. In cases where a traffic-duplicated uterus is present, as in the current case, menstrual blood drains via the left-right communication, often leading to a lack of obvious symptoms (Figure 5). In this case, the patient experienced normal menstrual bleeding after menarche, and apart from a cyst on the right vaginal wall, there were no noticeable subjective symptoms. This made diagnosis challenging due to the rarity of the condition.

While there have been sporadic reports of Herlyn-Werner syndrome, Wunderlich syndrome, and OHVIRA syndrome with the advent of imaging modalities such as MRI and CT scans, there is a scarcity of reports specifically addressing pregnancy and perinatal outcomes in this group of patients. Although there are indications that these syndromes may have some impact on fertility [8], their perinatal management requires careful consideration. Zhu et al. observed that in patients with Herlyn-Werner-Wunderlich syndrome, 64% of pregnancies occurred in healthy uteri, while 37% occurred in affected uteri [9]. Additionally, Dabi et al. reported a 17.4% infertility rate in individuals who underwent surgery for a duplicated uterus and closed vagina [10]. Moreover, in terms of perinatal consequences, elevated rates of miscarriage (74%), preterm delivery (22%), and cesarean sections (80%) have been documented [11,12], underscoring the need to consider the increased risks of miscarriage and preterm delivery when managing this group of disorders.

In the current case, the patient had a history of treatment for a cyst on the right vaginal wall over a decade ago. However, a conclusive diagnosis regarding whether this cyst represented a blind-angle uterine tear, a Gartner's duct cyst, or a blind-angle vaginal tear could not be made due to the absence of histological investigation. Nevertheless, the combination of features, including a duplicated uterus, paracervical cyst, and ipsilateral renal aplasia, strongly suggests Wunderlich syndrome or OHVIRA syndrome. As the initial examination did not yield a diagnosis beyond a bicornuate uterus, and the patient had a prior post-cesarean pregnancy, the possibility of an impending uterine rupture took precedence over the diagnosis of an incompletely occluded duplicated uterus. This was especially true considering the hypoechoic area of the cervix adjacent to the omentum and the clinical symptoms. A basic CT scan, which revealed a right renal defect for the first time, facilitated the diagnosis of Wunderlich syndrome, Herlyn-Werner syndrome, or OHVIRA syndrome. Additionally, while the clinical symptoms closely resembled those of clinical chorioamnionitis, the amniotic fluid examination allowed for the exclusion of chorioamnionitis. Along with a straightforward MRI scan, we successfully diagnosed an infection in the non-pregnant side of the duplicated uterus and extended the gestation period through the administration of antibiotics.

Although a traffic-incomplete obstructed duplicated uterus does not show symptoms in the non-gravid state, the insights gained from this case highlight potential perinatal complications. When diagnosed during the non-pregnant period, considering non-pregnant uterine infection or Gartner's duct cyst infection becomes crucial in differentiating OHVIRA syndrome from chorioamnionitis in pregnant patients who present with signs of infection, as in this case. Notably, a documented case report describes favorable perinatal outcomes for both the mother and child through early intervention, which involved opening the vaginal septum and preventing miscarriage due to infection of the contents [13]. Therefore, when dealing with uterine malformations such as a bicornuate or duplicated uterus, or a vaginal wall cyst, it is vital to conduct concomitant ultrasonographic examinations to confirm the presence and condition of the kidneys. This allows for early diagnosis of this syndrome and may be instrumental in managing women's health, including during the perinatal period. It remains crucial to treat this rare condition with proper consideration for the associated diseases, even though it occurs infrequently.

## Conclusions

In cases of a communicating duplicated uterus, menstrual blood from the affected side is expelled through the connection between the two uterine cavities, often leading to a lack of obvious symptoms. However, complications can arise during the perinatal period, as demonstrated in this case. If identified outside of pregnancy, the presence of infection symptoms during pregnancy, as seen here, requires consideration of various diagnoses. These may include infection of the non-pregnant uterine cavity or infection of a Gartner duct cyst as potential causes of chorioamnionitis. Therefore, when diagnosing uterine anomalies such as a bicornuate or duplicated uterus, or vaginal wall cysts, it is crucial to also assess the presence of the kidneys using ultrasonography or similar imaging techniques. This approach allows for early detection of associated syndromes and proves beneficial in managing the perinatal period.
